# Prehospital infection control and prevention in Denmark: a cross-sectional study on guideline adherence and microbial contamination of surfaces

**DOI:** 10.1186/s13049-018-0541-y

**Published:** 2018-09-05

**Authors:** Heidi Storm Vikke, Matthias Giebner, Hans Jørn Kolmos

**Affiliations:** 1 Department of Clinical Research, University of Southern Denmark, Odense, Denmark; 2 Falck Denmark A/S, Kolding, Denmark; 3 A & E Department, Sygehus Soenderjylland, Aabenraa, Denmark; 4 Department of Clinical Microbiology, Odense University Hospital, Odense, Denmark

## Abstract

**Background:**

Prehospital acute care and treatment have become more complex, and while invasive procedures are standard procedures, focus on infection control and prevention is scarce. We aimed to evaluate guideline adherence, microbial contamination, and associated risk factors.

**Methods:**

In a nationwide cross-sectional study, we evaluated guideline adherence to thorough cleaning (TC) once a day, and moderate cleaning (MC) in-between patient courses. Microbial contamination on hand-touch sites (HTS) and provider-related sites (PRS) was assessed by total aerobic colony forming units (CFU) and presence of selected pathogens, using swab and agar imprints. Also, microbial contamination was assessed in relation to potential risk factors.

**Results:**

80 ambulances and emergency medical service (EMS) providers were enrolled. Adherence to guidelines regarding TC was 35%, but regarding MC it was 100%. In total, 129 (27%) of 480 HTS presented a total CFU > 2.5/cm^2^ and/or pathogenic growth, indicating hygiene failures. The prevalence of selected pathogens on HTS was: *S. aureus* 7%; *Enterococcus* 3% and *Enterobacteriaceae* 1%. Total CFU on the PRS ranged from 0 to 250/cm^2^, and the prevalence of pathogens was 18% (*S. aureus* 15%, *Enterococcus* 3% and *Enterobacteriaceae* 0.3%). Methicillin-resistant *S. aureus* was found in one sample*,* and Vancomycin-resistant *Enterococcus* in two. No *Enterobacteriaceae* with extended-spectrum beta-lactamases were recorded.

**Conclusion:**

Guideline adherence was suboptimal, and many HTS did not comply fully with proposed standards for cleanliness. Pathogens were demonstrated on both HTS and PRS, indicating that the EMS may be a source of infection in hospitalized patients. Moreover, cleaning effort and time appears associated with microbial contamination, but a comprehensive investigation of risk factors is needed.

## Background

Despite decades with a focus on infection control and prevention, the healthcare-associated infection prevalence is 5–10% in developed countries. These infections may imply prolonged hospital stay for patients, the risk of disability, and increased mortality. Further, they are a financial burden for the healthcare system [1]. Cleaning of surfaces can reduce the transmission of pathogens in healthcare settings [2]. Proposed standards to evaluate cleaning efficiency have been set up for hospitals, comprising a total aerobic colony forming units count of < 2.5 or 5 CFU per cm^2^ and < 1 CFU/cm^2^ of healthcare-associated pathogens (e.g., *Staphylococcus aureus, Enterococcus species,* etc.) [2, 3]. However, no such standards have been implemented in the emergency medical services (EMS). During the last 10–20 years, prehospital acute care and treatment have become more complex, and thus an increased number of invasive procedures such as intravenous medical treatment and intubation are being performed outside controlled hospital environments, involving a potential risk of infection [4, 5]. Numerous aspects challenge infection control and prevention in the EMS. The ambulances carry several patients during a day. Scarce information with little or no focus on infection status is given to the EMS providers before retrieval of the patient [6]. The ambulances are non-static environments that must be ready for service shortly after delivering the patient to the emergency department, hence offering limited time and equipment to clean and decontaminate [7]. In addition to logistic challenges, studies indicate that implementation of cleaning measures [7, 8] and efficiency of cleaning [9] within the EMS vary. Moreover, pathogens have been found in both the ambulances [6, 7, 10], on uniforms [11, 12] and on the hands of the providers [13], and the spread of microbes within the ambulance environment is a proven reality [9]. Revised and updated prehospital hygiene guidelines, published in Denmark in 2016, involve both cleaning and hand- and uniform hygiene [14]. However, current adherence to these guidelines is uncertain, since no evaluation has been conducted. Moreover, evidence of microbial contamination and potential risks is sparse.

In this study we aimed to 1) evaluate adherence with current guidelines regarding cleaning, 2) assess microbial contamination on hand-touch sites (HTS) and provider-related sites (PRS), and 3) identify potential risk factors related to microbial contamination.

## Methods

### Design and setting

A nationwide cross-sectional study was conducted in cooperation with four prehospital governmental organizations, from August to November 2016. The North Denmark Region is 7.874 km^2^ and has a population of 0.5 million, with around 50 ambulances responding to approximately 55.000 prehospital missions annually. The Central Denmark Region is 13.053 km^2^, has a population of 1.304 million and 66 ambulances responding to approximately 150.000 missions annually. The Region Zealand is 7.217 km^2^ with a population of 0.8 million and 66 ambulances responding to approximately 66.000 missions annually. The Capital Region of Denmark is 2.546 km^2^, with a population of 1.822 million and 79 ambulances responding to approximately 190.000 missions annually.

### Danish prehospital hygiene guidelines

The guidelines impose thorough cleaning (TC) of the ambulance once a day, including tidying up in the patient compartment, overall surface wiping, and sweeping and washing the floor. Water and universal soap are the first choice but should be supplemented by disinfectant wipes with ethanol (70%) if there is a risk of contamination with body fluids. If the equipment does not tolerate water, disinfectant wipes with ethanol (70%) may be used instead. In addition to TC, moderate cleaning (MC) must be conducted after every patient course. This procedure includes tidying up in the patient compartment and wiping off HTS, using pre-soaked wipes containing a detergent (ex. Cocamidopropyl PG-Dimonium Chloride Phosphate, Sodium Benzoate, Potassium Sorbate, Lactic Acid), ethanol (70%), or bleach (1000–1200 ppm) depending on risks related to the previous patient course. All cleaning procedures are conducted by EMS providers. In addition to the cleaning measures, the providers are expected to begin the shift wearing a clean uniform, and to act in accordance with hand hygiene guidelines, comprising hand rub before and after patient contact, if hands are not visibly soiled. If hands are visibly soiled, hand wash should be conducted before hand rub (if water and soap are not available, pre-soaked wipes for skin care may be used). Moreover, the use of examination gloves is recommended if there is a risk of body fluid contamination, e.g., blood, mucus, feces or urine, or if the patient’s infection status demands special preventive precautions [14].

### Selection

Our goal was to enroll 80 ambulances and providers, 20 from each of the enrolled regions. Four emergency departments were designated as enrolment locations, each with one unannounced sampling date. The person responsible for microbial sampling and collection of informative data (first author) was present at each emergency departments’ ambulance arrival area from 7 am to 7 pm, on the four days of collection. All EMS providers were informed about the study purpose and asked for participating consent shortly after patient delivery and MC.

### Collection of data on cleaning adherence

The providers were asked if they had conducted TC during their shift, and adherence to guidelines regarding MC was registered if it was conducted.

### Registration of potential risk factors

Time of sampling was registered, and the providers were asked about the number of patient courses completed during the shift. Also, they were asked about the location of the ambulance branch (name of the city), and area of service was later defined as city if the branch was located in an area with more 40.000 citizens, and as rural, if it was located in an area with less than 40.000 citizens.

### Evaluation of microbial contamination

Comparable to prior mentioned proposed standards for cleanliness in healthcare settings [2, 3] we chose to define a site “clean” if total CFU ≤ 2.5/cm^2^ and no selected pathogens were detected. Our focus included the following pathogens: *Staphylococcus aureus*, *Enterococcus species* and *Enterobacteriaceae* (*E. coli, Klebsiella pneumoniae, Proteus mirabilis, Citrobacter koseri* and *Enterobacter cloacae)*. Within each of these species we looked specifically for the following resistant organisms: Methicillin resistant *Staphylococcus aureus* (MRSA), Vancomycin-resistant enterococci (VRE), and *Enterobacteriaceae* with extended spectrum beta-lactamases (ESBL).

### Test sites

Based on prior EMS findings indicating hygienic challenges in relation to HTS [7, 15], we chose to sample the patient harness, the handle on the medic bag, the front of the defibrillator, the inside the blood pressure cuff, the handlebar in ambulance ceiling, and the wall next to the patient stretcher. To elucidate the level of microbial contamination on PRS, we sampled the torso and sleeve from the uniforms [11] and the upper side, and the palm of the dominating hand from the provider last in charge of a patient course [13].

### Sampling and processing

The samples were collected combining two methods. We chose to use Dip Slides (Plate count/VRBG agar incl. Neutralizers, VWR - Bie & Berntsen A/S, DK) and Flocked Swabs (eSwab™, SSI Diagnostica, DK) to secure recovery abilities and technical simplicity [3, 16].

Imprints were obtained holding a Dip Slide on the site for 10 s, with a pressure of approximately 25 g/cm^2^. The Dip Slides were kept cold until they were incubated aerobically for 48 h at 36C^o^. After incubation, total CFU/cm^2^ was determined by calculating the colonies on the plate count side and classifying each result either 0, 2.5, 12, 40, 100 or 250, as recommended by the manufacturer key. Growth on the VRBG side (*Enterobacteriaceae* specific) was visually inspected, and present colonies were transferred onto blood agar (Columbia agar + 5% sheep blood, Biomerieux, DK) and incubated for 24 h at 36C^o^ initial to Matrix-Assisted Laser Desorption/Ionization-Time of Flight Mass Spectrometry (MALDI-TOF MS) identification.

The swab samples were retrieved from a 5-cm^2^ area, next to were the dip slide imprint had been taken, moving a Liquid Amies moistened Flocked swab (dipped in the medium) in two directions at right angles in a zigzag pattern while rotating the swab. The samples were kept cold until inoculation in serum broth and 18 h of propagation at 36C^o^. After propagation, 2 × 25 μl of the sample was transferred onto CPS/CNA agar plates (Chromid Cps Elite/Columbia Cna 5% sheep blood, Biomerieux, DK) and incubated aerobically for 48 h at 36C^o^. Growth was then identified by inspection and microscopy. Suspected *S. aureus*, *Enterococcus* or *Enterobacteriaceae* (*E. coli, Klebsiella pneumoniae, Proteus mirabilis, Citrobacter koseri* and *Enterobacter cloacae)* were transferred onto blood agar plates (Columbia agar + 5% sheep blood, Biomerieux, DK) and incubated for another 24 h at 36C^o^ before final identification using MALDI-TOF MS.

The MALDI-TOF MS identification process in brief: colonies from the blood agar plates were transferred onto a target steel plate, alfa-cyano-matrix was applied, and the target plate allowed to dry before being placed in the Biotyper (Bruker Microflex LT, Germany). Identification-scores > 1.7 were considered valid.

Antibiotic resistance of pathogens within the scope of our study was determined by disc diffusion, in accordance with local clinical laboratory standards (Department of Clinical Microbiology, Odense University Hospital, Denmark).

All sampling, processing, and registration were conducted by one individual (first author) to secure rigidity and precision. Furthermore, all laboratory findings were reviewed by an experienced laboratory technician.

### Statistical analyses

Data was registered by paper and entered STATA 14 for analysis. To prevent errors, each variable was compared to pre-defined expected values, by running the STATA codebook-command. Descriptive statistics were conducted, and the median, 25th and 75th percentiles and range were calculated for the ordinal variable; total CFU. For the numeric variable; number of patients, mean and range were calculated. For the binary variables; presence of pathogens (yes/no), area of service (city/rural) and TC status (conducted/omitted) we calculated frequencies and proportions. Analysis of total CFU by TC status was conducted using the Wilcoxon rank-sum test for each site, respectively. Whereas, the analysis of pathogen presence by TC status was conducted using the Two-sample test of proportion for each site, respectively. Initial to the analysis of potential risk factors related to microbial contamination we assessed the correlation between the numerical variables; time of sampling and number of patients using the Spearman’s row test (due to non-normality). Using logistic regression, we assessed the potential risk related to a site not being clean (a site presenting a total CFU > 2.5/cm^2^ and/or growth of pathogens). The variable; number of patients was omitted from the risk factor analysis due to strong correlation with the variable; time of sampling (Spearman’s rho: 0.69 *p* = 0.000). The results of the risk factor analysis were reported by odds ratios (OR) and 95-confidence intervals (95%CI). Alpha was 0.05 in all the analyses.

## Results

We enrolled 80 ambulances and coherent EMS providers, and thus retrieved a total of 800 samples during four days of data collection (from August to November 2016). In total, 38 (48%) of the enrolled ambulances were operating in a city area and 42 (52%) in a rural area. The average ambulance and coherent providers had completed two patient courses by the time of sampling (Range 0–6).

### Guidelines adherence

As to adherence to cleaning, we found that TC had been conducted in 28 (35%) of the 80 enrolled ambulances. Whereas, MC was registered in all 80 (100%) ambulances.

### Assessment of microbial contamination on hand-touch sites (HTS)

In total, 93 (19%) of the 480 sites presented a total CFU > 2.5/cm^2^. Median total CFU was 2.5/cm^2^ on all sites, respectively. Total CFU ranged highest on the blood pressure cuffs, the medic bag handles and on the patient harnesses’ (Table 1).

**Table 1:** Total aerobic colony forming units (CFU)/cm^2^, regardless of thorough cleaning (TC)

Test sites	n	Median	25p-75p	Range
Medic bag handle	80	2.5	2.5–12	0–40
Patient harness	80	2.5	2.5–2.5	0–40
Blood pressure cuff	80	2.5	2.5–12	0–100
Defibrillator	80	2.5	2.5–2.5	0–12
Patient near site	80	2.5	2.5–2.5	0–12
Handlebar ceiling	80	2.5	1.25–2.5	0–12
Uniform torso	80	12	2.4–40	0–250
Uniform sleeve	80	12	2.5–40	0–250
Upper hand	80	2.5	2.5–12	0–250
Palm of hand	80	2.5	2.5–12	0–40

Pathogens were recovered from 49 (10%) of the 480 sites (95%CI 8–13), and the prevalence varied in relation to site origin and species. *S. aureus* was recovered from 7%, *Enterococcus* from 3%, and *Enterobacteriaceae* from 1%. Similar to what we found regarding total CFU, the prevalence of pathogens was highest among the blood pressure cuffs, the medic bag handles and the patient harnesses (Table 2).

**Table 2:** Prevalence of pathogens, regardless of thorough cleaning (TC)

Test sites	n	% (95% CI)
Medic bag handle	80	14 (8–23)
Patient harness	80	13 (7–22)
Blood pressure cuff	80	18 (11–28)
Defibrillator	80	6 (3–14)
Patient near site	80	10 (5–19)
Handlebar ceiling	80	1 (0–8)
Uniform torso	80	24 (16–34)
Uniform sleeve	80	21 (14–32)
Upper hand	80	13 (7–22)
Palm of hand	80	16 (10–26)

**Fig. 1:**
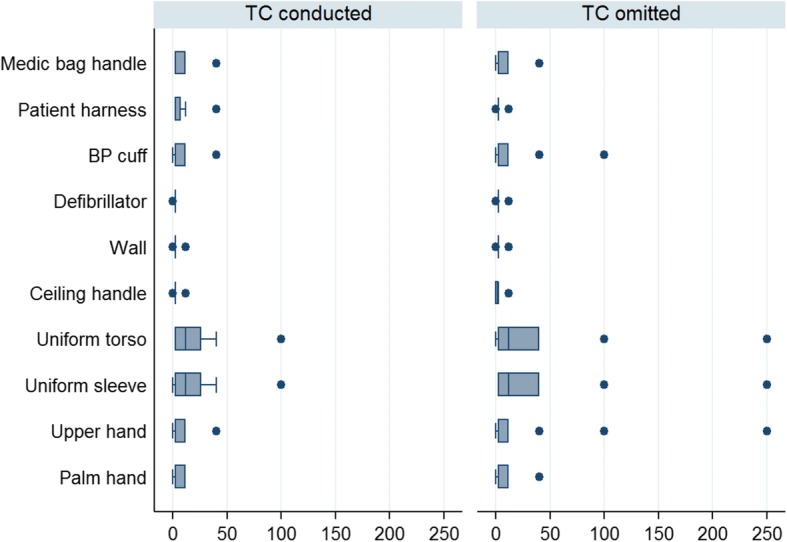
Total colony forming units (CFU)/cm^2^ per site, according to thorough cleaning (TC) status

**Fig. 2:**
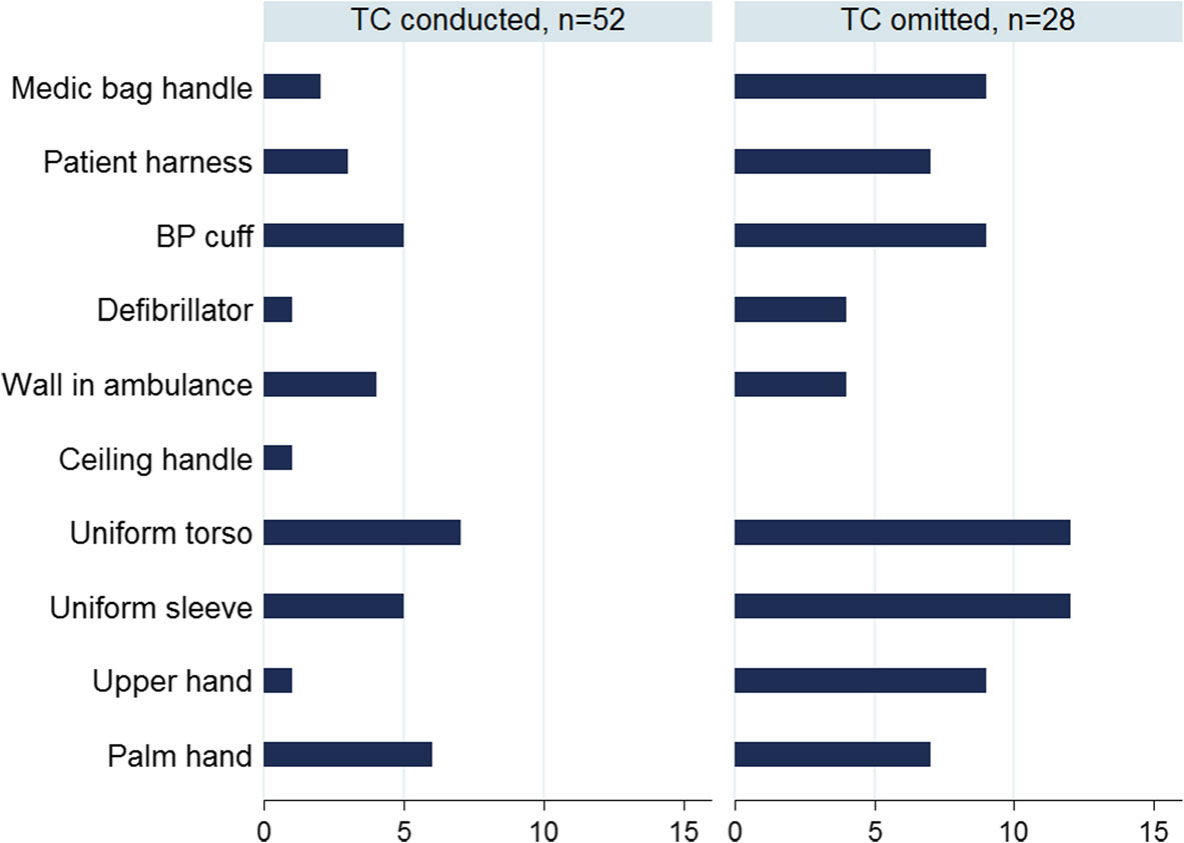
Frequency of sites presenting pathogens per site, according to thorough cleaning (TC) status

Moreover, there was a tendency towards more profound contamination on HTS when TC had been omitted; however, it did not reach significance (Fig. 1. Total colony forming units (CFU)/cm^2^ per site, according to TC status, and Fig. 2. Frequency of sites presenting pathogens per site, according to TC status).

### Assessment of microbial contamination on EMS providers

The median total CFU on the uniforms and hands were 12 and 2.5/cm^2^, respectively, and the range varied from 0 to 250 CFU/cm^2^. The growth of pathogens was demonstrated in 59 (18%) of the samples and varied in relation to site origin and species (Table 2). *S. aureus* was found on 48 (15%), *Enterococcus* on 10 (3%) and *Enterobacteriaceae* on 1 (0.3%). Once again, neither total CFU nor growth of pathogens did vary significantly by TC status, but we did detect a tendency towards more profound contamination if TC had been omitted (Fig. 1. Total CFU/cm^2^ per site, according to TC status, and Fig. 2. Frequency of pathogens per site, according to TC status).

### Microbes with resistance properties

Of the 108 sites positive for pathogens, one *Staphylococcus aureus* was Methicillin-resistant (MRSA), and two *Enterococcus* were Vancomycin-resistant (VRE). *Enterobacteriaceae* with extended-spectrum beta-lactamases (ESBL) were not recovered.

### Potential risk factors associated with microbial contamination

Overall, 129 (27%) of the 480 HTS and 190 (59%) of the PRS were not clean, and thus presented a total CFU > 2.5/cm^2^ and/or growth of pathogens, but the contamination was not significantly associated to any of the risk factors assessed (Table 3).

**Table 3:** Potential risk factors associated with sites not being clean (CFU > 2.5/cm^2^ and/or growth of pathogens)

Risk factors	Univariate analysis		Multivariate analysis	
	OR (95% CI)	*P*	OR (95% CI)	*P*
HTS, *n* = 480				
Time of day of sampling^a^	0.9 (1.0–1.2)	0.072	0.9 (0.9–1.0)	0.068
Rural service	1.0		1.0	
City service	1.0 (0.7–1.5)	0.955	1.1 (0.7–1.6)	0.773
Provider-related sites, *n* = 320				
Time of day of sampling^a^	1.0 (0.9–1.1)	0.738	0.9 (0.9–1.1)	0.707
Rural service	1.0		1.0	
City service	1.0 (0.7–1.6)	0.864	1.1 (0.7–1.7)	0.810

^a^Total sampling period: 07.00 am - 06.00 pm. on the four days of data collection. Time of sampling classified by 1-h intervals, e.g., samples collected from 07.00 am - 08.00 am, classified as 07.00 am, samples collected from 08.00–09.00 am as 08.00, etc

However, there was a tendency towards the time of day of sampling being associated with a reduced risk of a HTS not being clean (OR 0.9, CI 95%: 0.9–1.0, *p* = 0.068).

## Discussion

TC had been conducted in 35% of the ambulances, and MC in 100%. In total, 27% of the HTS were not clean and thus presented a total CFU > 2.5/cm^2^ and/or growth of pathogens. Total CFUs on the PRS ranged from 0 to 250/cm^2^, and the prevalence of pathogens was 18%. Overall, we found MRSA in one sample, and VRE in two, but no ESBL were recorded. Also, we found a tendency towards time of day of sampling lowering the risk of a site not being clean, which makes sense given that time of sampling was associated with number of patient courses, and thus also with the number of MC procedures performed.

Cleaning in healthcare settings is one of the cornerstones of infection control and prevention [8] and emphasized in the Danish prehospital hygiene guidelines [14]. Nevertheless, we discovered a suboptimal adherence to daily TC, which could be related to various factors, e.g., time pressure, lack of prioritization and/or insufficient education [8, 17]. However, further research is needed to investigate these assumptions.

The level of contamination on the HTS in our study is similar to prior findings [6, 15], but the prevalence of resistant bacteria [7] is lower. The latter reflects the generally low level of resistance seen in Denmark [18]. Detecting a level of microbial contamination above proposed standards despite cleaning is a prior documented EMS problem [15, 17], and thus we add to the evidence on challenges related to cleaning efficiency. Explanations for compromised cleaning efficiency could be identical to the ones influencing the guideline adherence, but further research is required to draw any firm conclusions.

Several cleaning and disinfection procedures have been evaluated in the EMS setting, e.g., conventional cleaning and secondary disinfection [9], fumigation [19, 20] and ultraviolet germicidal irradiation [21], but all came out either sub-efficient or very time consuming (> 1 h), leaving that problem unresolved.

The contamination on the uniforms found in the present study is in line with prior findings [11, 12], and thus supports the need for a daily change of EMS uniforms and efficient washing procedures. The Danish prehospital hygiene guidelines recommend that uniforms are washed at 80C after every shift [14], but the high temperature is incompatible with the fact that the traffic reflectors on the EMS uniforms (protecting the providers when working at dark roadsides) do not tolerate high temperatures. However, domestic washing at 60C using a detergent containing *acetic peroxide*, supplemented by tumble drying has shown a potential to eliminate pathogens when applied in an EMS setting [11] and could thus represent a good alternative.

Finally, profound microbial contamination on the hands of EMS providers has been documented by Teter et al. [13], and thus our results add to the concern of EMS hands as vectors for transmission of pathogens. Such concern is emphasized by suboptimal hand hygiene compliance among EMS providers [17, 22].

### Strengths and limitations

To the best of our knowledge, this study is novel to conduct a comprehensive investigation of prehospital infection control and prevention in the Scandinavian setting, and it underlines the relevancy in highly developed EMS settings. Nevertheless, our study has some limitations. First, the ambulances and providers were not sampled multiple times during the same day, and thus we are unable to clarify the exact progress in microbial contamination. Moreover, we only retrieved samples after MC had been conducted, and thus we are unable to elucidate the precise effect of MC. Finally, we did not determine the pathogens at a genomic level, and thus we are unable to assess coherence between microbes on HTS and PRS. Also, we acknowledge a risk of self-reporting bias occurring because the providers were asked if TC had been conducted. Their answers may have been affected by social desirability or approval, leading to an overestimation of the guideline compliance, especially because TC was regarded as mandatory in the organization. Unfortunately, we did not have any other way to retrieve information about TC status.

## Conclusions

In conclusion, our study shows that adherence to cleaning practices is far from optimal in the EMS. As a consequence, contamination of surfaces often exceeds levels proposed in standards for other health-care settings. Previous studies have shown that infectious microbes can be spread in ambulances [9] and that the dose necessary for causing infections in patients is low [23]. Our results, therefore, call for concern and the need for improvement. Future studies should focus on how to increase compliance with proposed standards. Further, there is a need for studies that more in detail define acceptable levels of contamination in the EMS.

## Abbreviations

CFU: Colony forming units
EMS: Emergency medical service
ESBL: Enterobacteriaceae with extended spectrum beta-lactamases
HTS: Hand-touch site
MC: Moderate cleaning
MRSA: Methicillin resistant *Staphylococcus aureus*
PRS: Provider-related site
TC: Thorough cleaning
VRE: Vancomycin-resistant Enterococci

## References

[1] World Health Organization (WHO). Report on the Burden of Endemic Health Care-Associated Infection Worldwide A systematic review of the literature 2011. Available from: Http://apps.who.int/iris/bitstream/10665/80135/1/9789241501507_eng.pdf?ua=1 (Accessed: 24/06/2018).

[2] Dancer SJ (2014). Controlling hospital-acquired infection: focus on the role of the environment and new technologies for decontamination. Clin Microbiol Rev.

[3] Dancer SJ (2004). How do we assess hospital cleaning? A proposal for microbiological standards for surface hygiene in hospitals. J Hosp Infect.

[4] Ryynänen O-P, Iirola T, Reitala J, Pälve H, Malmivaara A (2010). Is advanced life support better than basic life support in prehospital care? A systematic review. Scandinavian Journal of Trauma, Resuscitation and Emergency Medicine.

[5] Williamson K, Ramesh R, Grabinsky A (2011). Advances in prehospital trauma care. International Journal of Critical Illness and Injury Science.

[6] Luksamijarulkul P, Pipitsangjan S (2015). Microbial air quality and bacterial surface contamination in ambulances during patient services. Oman medical journal.

[7] Wepler M, Stahl W, von Baum H, Wildermuth S, Dirks B, Georgieff M, Hafner S (2015). Prevalence of nosocomial pathogens in German ambulances: the SEKURE study. Emerg Med J.

[8] Bledsoe BE, Sweeney RJ, Berkeley RP, Cole KT, Forred WJ, Johnson LD (2014). EMS provider compliance with infection control recommendations is suboptimal. Prehospital Emergency Care..

[9] Valdez MK, Sexton JD, Lutz EA, Reynolds KA (2015). Spread of infectious microbes during emergency medical response. Am J Infect Control.

[10] Rago JV, Buhs LK, Makarovaite V, Patel E, Pomeroy M, Yasmine C (2012). Detection and analysis of Staphylococcus aureus isolates found in ambulances in the Chicago metropolitan area. Am J Infect Control.

[11] Vikke HS, Giebner M (2015). UniStatus-a cross-sectional study on the contamination of uniforms in the Danish ambulance service. BMC research notes.

[12] Groß R, Hübner N, Assadian O, Jibson B, Kramer A, Hygiene WSfCAotGSfH. Pilot study on the microbial contamination of conventional vs. silver-impregnated uniforms worn by ambulance personnel during one week of emergency medical service GMS Krankenhaushygiene interdisziplinär. 2010;5(2). 10.3205/dgkh000152 PMC2951103 20941337

[13] Teter J, Millin MG, Bissell R (2015). Hand hygiene in emergency medical services. Prehospital emergency care : official journal of the National Association of EMS Physicians and the National Association of State EMS Directors.

[14] Statens Serum Institut (SSI). Nationale Infektionshygiejniske Retningslinjer. 2016. Available from: https://www.ssi.dk/~/media/Indhold/DK%20-%20dansk/Smitteberedskab/Infektionshygiejne/NIR/NIR%20praehospital.ashx (Accessed: 30/05/2018).

[15] Vikke HS, Giebner M (2016). POSAiDA: presence of Staphylococcus aureus/MRSA and enterococcus/VRE in Danish ambulances. A cross-sectional study. BMC Research Notes.

[16] Dalmaso G, Bini M, Paroni R, Ferrari M (2008). Qualification of high-recovery, flocked swabs as compared to traditional rayon swabs for microbiological environmental monitoring of surfaces. PDA J Pharm Sci Technol.

[17] Bucher J, Donovan C, Ohman-Strickland P, McCoy J (2015). Hand washing practices among emergency medical services providers. West J Emerg Med.

[18] DANMAP 2016 - Use of antimicrobial agents and occurrence of antimicrobial resistance in bacteria from food animals, food and humans in Denmark. Available from: https://www.danmap.org/~/media/Projekt%20sites/Danmap/DANMAP%20reports/DANMAP%202016/DANMAP_2016_web.ashx (Accessed: 30/05/2018).

[19] Alrazeeni D, Al Sufi MS (2014). Nosocomial infections in ambulances and effectiveness of ambulance fumigation techniques in Saudi Arabia. Phase I study Saudi medical journal.

[20] Lowe JJ, Hewlett AL, Iwen PC, Smith PW, Gibbs SG (2013). Evaluation of ambulance decontamination using gaseous chlorine dioxide. Prehospital Emergency Care.

[21] Lindsley WG, McClelland TL, Neu DT, Martin SB, Mead KR, Thewlis RE, Noti JD (2018). Ambulance disinfection using ultraviolet germicidal irradiation (UVGI): effects of fixture location and surface reflectivity. J Occup Environ Hyg.

[22] Emanuelsson L, Karlsson L, Castrèn M, Lindström V (2013). Ambulance personnel adherence to hygiene routines: still protecting ourselves but not the patient. Eur J Emerg Med.

[23] Otter JA, Yezli S, French GL (2011). The role played by contaminated surfaces in the transmission of nosocomial pathogens. Infect Control.

[24] Ministry of Higher Education and Science. The Danish Code of Conduct for Research Integrity. Available from: https://ufm.dk/publikationer/2014/filer-2014/the-danish-code-of-conduct-for-research-integrity.pdf (Accessed: 30/05/2018).

[25] The World Medical Association, INC, Declaration Of Helsinki, Ethical principles for Medical Research Involving Human Subjects. Available from: https://www.wma.net/wp-content/uploads/2016/11/DoH-Oct2008.pdf (Accessed: 30/05/2018).

